# Effect of Monomer Type on the Synthesis and Properties of Poly(Ethylene Furanoate)

**DOI:** 10.3390/polym15122707

**Published:** 2023-06-16

**Authors:** Johan Stanley, Zoi Terzopoulou, Panagiotis A. Klonos, Alexandra Zamboulis, Eleftheria Xanthopoulou, Savvas Koltsakidis, Dimitrios Tzetzis, Lidija Fras Zemljič, Dimitra A. Lambropoulou, Apostolos Kyritsis, George Z. Papageorgiou, Dimitrios N. Bikiaris

**Affiliations:** 1 Laboratory of Chemistry and Technology of Polymers and Colors, Department of Chemistry, Aristotle University of Thessaloniki, GR-541 24 Thessaloniki, Greece; johansta@chem.auth.gr (J.S.); terzozoi@chem.auth.gr (Z.T.); panos48al@gmail.com (P.A.K.); azampouli@chem.auth.gr (A.Z.); 2 Department of Physics, Zografou Campus, National Technical University of Athens, 15780 Athens, Greece; akyrits@central.ntua.gr; 3 Department of Chemistry, University of Ioannina, GR-45110 Ioannina, Greece; elefthxanthopoulou@gmail.com (E.X.); gzpap@uoi.gr (G.Z.P.); 4 Digital Manufacturing and Materials Characterization Laboratory, School of Science and Technology, International Hellenic University, 14 km Thessaloniki, 57001 N. Moudania, Greece; skoltsak@meng.auth.gr (S.K.); d.tzetzis@ihu.edu.gr (D.T.); 5 Faculty of Mechanical Engineering, University of Maribor, SI-2000 Maribor, Slovenia; lidija.fras@um.si; 6 Laboratory of Environmental Pollution Control, Department of Chemistry, Aristotle University of Thessaloniki, GR-541 24 Thessaloniki, Greece; dlambro@chem.auth.gr; 7 Center for Interdisciplinary Research and Innovation (CIRI-AUTH), Balkan Center, GR-570 01 Thessaloniki, Greece; 8 Institute of Materials Science and Computing, University Research Center of Ioannina (URCI), 45110 Ioannina, Greece

**Keywords:** bio-based polymers, poly(ethylene furanoate), polycondensation, thermal properties, mechanical properties, oxygen transmission rates

## Abstract

This work aimed to produce bio-based poly(ethylene furanoate) (PEF) with a high molecular weight using 2,5-furan dicarboxylic acid (FDCA) or its derivative dimethyl 2,5-furan dicarboxylate (DMFD), targeting food packaging applications. The effect of monomer type, molar ratios, catalyst, polycondensation time, and temperature on synthesized samples’ intrinsic viscosities and color intensity was evaluated. It was found that FDCA is more effective than DMFD in producing PEF with higher molecular weight. A sum of complementary techniques was employed to study the structure–properties relationships of the prepared PEF samples, both in amorphous and semicrystalline states. The amorphous samples exhibited an increase in glass transition temperature of 82–87 °C, and annealed samples displayed a decrease in crystallinity with increasing intrinsic viscosity, as analyzed by differential scanning calorimetry and X-ray diffraction. Dielectric spectroscopy showed moderate local and segmental dynamics and high ionic conductivity for the 2,5-FDCA-based samples. The spherulite size and nuclei density of samples improved with increased melt crystallization and viscosity, respectively. The hydrophilicity and oxygen permeability of the samples were reduced with increased rigidity and molecular weight. The nanoindentation test showed that the hardness and elastic modulus of amorphous and annealed samples is higher at low viscosities due to high intermolecular interactions and degree of crystallinity.

## 1. Introduction

With the increase in the global population to 8 billion, plastic contaminants and pollutants significantly impact the environment, which should be a serious consideration for the earth’s future. Using petroleum-derived polymers causes enormous damage to our ecosystem and people’s health. Poly(ethylene terephthalate) (PET) is one of the widely used synthetic polymers in the food packaging industry (to produce bottles, wide mouth jars, tubs, trays, films, and coatings) due to its good barrier, thermal and mechanical properties [1]. However, recent studies have shown that phthalate esters are found in water, stored in PET bottles for prolonged times at high temperatures, and directly exposed to sunlight. The leaching of phthalate esters from PET bottles can affect the endocrine system. Therefore, it increases the need to assess bottled water to be aware of the potential health risks of phthalate esters [2]. Other fossil-based polymers such as low-density polyethylene (LDPE), high-density polyethylene (HDPE), polypropylene (PP), and polystyrene (PS) are also widely used in the field of food packaging. More studies have been performed for producing fossil-based polymer nanocomposites to enhance the barrier and antimicrobial properties to be used as reactive packaging materials for food preservation [3]. In recent years, the efforts to replace petroleum-based polymers with bio-based polymers have been more intense for various applications to produce eco-friendly and user-friendly materials [4]. Bio-based polymers have emerged as environmentally friendly packaging materials, films, and coatings. However, the films and coatings made from bio-based polymers lack barrier and mechanical properties compared with petroleum-based polymers [5].

Poly(ethylene furanoate) (PEF) is a promising bio-based polyester synthesized from FDCA, or its derivate DMFD, and ethylene glycol (EG) with a superior barrier and thermal and mechanical properties to those of PET. The 100% renewable PEF offers a promising alternative to fossil-based commodity plastics in food packaging, such as bottles and films. PEF polyesters have a six-times-better O_2_ barrier, two-times-better H_2_O barrier, two- to four-times-better CO_2_ barrier, 1.6 higher tensile modulus, about 70% lower CO_2_ emission, and about 65% lower non-renewable energy use for PEF production. Most importantly, the FDCA monomer used for PEF production is generated from biomass. FDCA-based polyesters have drawn attention in the packaging industry due to their ability to formulate into films, fibers, and, mostly, bottles [6]. PEF has a glass transition temperature (*T_g_*) that is nearly 15 °C higher (85–89 °C vs. 70 °C) and a melting range that is 40 °C lower (210–215 °C for PEF vs. 250–260 °C for PET) than PET [7]. Therefore, it requires lower energy during melting processing and can be used at higher temperatures [8]. PEF polyesters generally have a large nucleating density and small spherulites even at higher crystallization temperatures.

PEF has a spiral motif and a nonlinear architecture because of the inherent angle (129.4°) between the C-C bonds of the carbonyl groups of FDCA. The spiral motif confers unique properties on the polyester, such as high stiffness, chemical stability, and excellent barrier properties [7,9]. The U.S. Department of Energy has determined that FDCA is one of the most important top value-added compounds produced from biomass [10]. The following methods are used to produce FDCA: (1) oxidizing aldehydes and the methylol group of 5-hydroxymethylfurfural (HMF) to carboxyl groups using chemical or biological processes, (2) converting 2-furoic acid into FDCA through the well-known Henkel reaction, (3) cyclodehydrating hexaric acid, and (4) cyclodehydration of glucose or fructose. Additionally, much research has been undertaken to produce FDCA using green methodology. Zhao et al. studied the greener synthesis method to produce diethyl furan-2,5-dicarboxylate (DEFDC) starting from mucic acid in ethanol via a one-pot, two-step procedure. The purified diethyl ester DEFDC and ethylene glycol were heated at 150 °C for two hours to produce prepolymer bis(hydroxyethyl)-2,5-furandicarboxylate (BHEFDC) using cobalt (II) acetate (Co(Ac)_2_·4H_2_O) as a catalyst. Finally, polyethylene-2,5-furan dicarboxylate was produced from BHEFDC prepolymer and one equivalent of DEFDC via solvent-free Co(Ac)_2_·4H_2_O catalyzed poly transesterification at 160 °C for 3 h [11]. Even though HMF is unstable as an intermediate product in acidic environments and a few by-products are inevitable, this method is still one of the most promising for the industrialization of FDCA [12]. As of yet, considerable attention has been paid only to the preparation of HMF, and several international companies such as Avantium, ADM, Shell, and BASF, have been involved with pilot-scale production [13].

**Figure 1:**
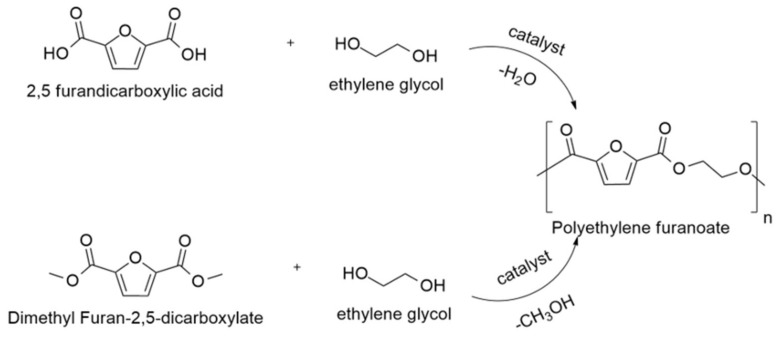
Synthesis route of PEF, starting from FDCA and DMFD.

Many researchers aim to produce high-molecular-weight PEF with reduced polycondensation time to minimize the influence of FDCA monomer on the color of PEF. First, FDCA is converted into DMFD via esterification with methanol under acidic conditions. Further, DMFD and EG undergo polycondensation to produce PEF using an efficient, colorless, and stable catalyst system [14]. Companies such as DuPont and ADM esterify their FDCA to its dimethyl ester (DMFD) to resolve FDCA’s instability and prevent thermo-oxidative degradation of FDCA polymers at high temperatures [15]. The synthesis route of PEF using both FDCA and its derivative DMFD has been illustrated in Figure 1. Even though DMFD is used in the synthesis of PEF with lower polycondensation time and reduced coloration of the PEF sample, from an industrial point of view, FDCA is preferred over DMFD as a starting material since atom efficiency for the polymerization of FDCA to PEF is higher and the esterification reaction from FDCA to DMFD shall be bypassed [16]. Additionally, the existing PET production lines can be easily adapted from terephthalic acid to furan dicarboxylic acid as a starting monomer [17].

In this work, PEF was synthesized using FDCA or its derivative DMFD with different monomer molar ratios, processing temperatures, and catalysts via a two-stage melt polycondensation reaction, targeting materials for food packaging applications. The aim was to compare the effectiveness of FDCA and DMFD in the production of high-molecular-weight PEF and to determine the advantages and limitations of each one monomer. To evaluate this, all produced materials have been extensively characterized regarding their molecular weight, thermal properties, oxygen transmission rates and mechanical properties. To the best of our knowledge, the materials of various [*η*] are studied by a sequence of complementary methods for the first time.

## 2. Materials and Methods

### 2.1. Materials

2,5-Furan dicarboxylic acid (BioFDCA X000230-2003) was purchased from Corbion, (Gorinchem, The Netherlands). Ethylene glycol (anhydrous, 99.8%), titanium butoxide, antimony trioxide, and zinc acetate were purchased from Aldrich Co (London, UK). Zirconium(IV) isopropoxide was purchased from Alfa Aesar (Haverhill, MA, USA). Dimethyl 2,5-furandicarboxylate was synthesized from 2,5-FDCA and methanol. All other materials and solvents used were of analytical grade.

### 2.2. Synthesis of PEF Using Dimethyl 2,5-Furandicarboxylate (DMFD)

The PEF polyesters were synthesized using a two-stage melt polycondensation process (esterification and polycondensation). Different molar ratios of DMFD/ethylene glycol (EG) (1/2.2, 1/1.5, 1/1.2) were used for the synthesis of PEF. Zinc acetate (50 ppm), zirconium (IV) isopropoxide isopropanol complex (400 ppm), and titanium butoxide (TBT) (400 ppm) were used as catalysts, as shown in Table 1. The apparatus containing the reagents was repeatedly evacuated and filled with nitrogen to remove the air completely. Under the nitrogen atmosphere, the reaction mixture was heated while being stirred continuously (200 rpm). The temperatures and time for the first step (transesterification) are displayed in Table 1. After extracting the entire theoretical amount of CH_3_OH, which was obtained from the reaction mixture by distillation, the transesterification step was considered completed. During the second step (polycondensation), a vacuum (5.0 Pa) was applied slowly over 15 min to remove excess diols, prevent excessive foaming, and, mainly, reduce oligomer sublimation. The different temperatures and times used for the second step (polycondensation) are given in Table 1. The reaction mixture was stirred continuously (100–150 rpm) under vacuum. The polyesters were extracted when the polycondensation reaction was finished, were milled, and were washed with methanol.

### 2.3. Synthesis of Polyester Using 2,5-Furandicarboxylic Acid (FDCA)

In the first step (esterification), FDCA and EG were used in a 1:2.1 molar ratio. Initially, the flask was evacuated and filled with nitrogen three times to remove the air completely. Next, the reaction mixture was pre-heated at 170 °C for 30 min and 190–200 °C for 1 h under nitrogen flow and with stirring speed at 200 rpm. Typically, within the first 1–1.5 h of the 1st step, H_2_O distillation occurs. After 1.5 h of heating, antimony trioxide (Sb_2_O_3_) (300 ppm) catalyst was added into the polymerization reactor and vacuum (5.0 Pa) was progressively implemented over 15 min. Furthermore, the temperature was gradually increased to 250–260 °C, and heating was continued for different times under vacuum (Table 1). At the same time, the stirring speed decreased (100–70–50 rpm) to avoid high shear stress. Finally, the samples were retrieved from the reactor, and characterized [16].

### 2.4. Characterization

#### 2.4.1. Nuclear Magnetic Resonance (NMR)

Nuclear magnetic resonance (NMR) spectra were recorded on an Agilent 500 spectrometer (Agilent Technologies, Santa Clara, CA, USA) and calibrated using the residual CDCl_3_ solvent peaks. PEF samples were dissolved in deuterated chloroform/deuterated trifluoroacetic acid mixtures. A dimethyl sulfoxide probe was also used for lock purposes for the ^13^C NMR spectra.

#### 2.4.2. Attenuated Total Reflection (ATR)

ATR spectra of the samples were recorded utilizing an IRTracer-100 (Shimadzu, Japan) equipped with a QATR™ 10 Single-Reflection ATR Accessory with a Diamond Crystal. The spectra were collected from 450 to 4000 cm^−1^ at a resolution of 2 cm^−1^ (total of 16 co-added scans), while the baseline was corrected and converted into absorbance mode.

#### 2.4.3. Intrinsic Viscosity Measurement

Intrinsic viscosity, [*ղ*], measurements were performed using an Ubbelohde viscometer (Schott Gerate GMBH, Hofheim, Germany) at 30 °C in a mixture of phenol/1,1,2,2-tetrachloroethane at a ratio of 60/40 *w*/*w*. Sample concentrations of 1% (*w*/*v*) were used. The [*ղ*] value of each sample was calculated using the following Solomon–Ciuta Equation (1):

(1) $$\left[\eta \right]=\frac{{[2\{\frac{t}{t_{0}}-\ln\left(\frac{t}{t_{0}}\right)-1\}]}^{\frac{1}{2}}}{c}$$

where *c* is the concentration of the solution, *t*_0_ is the flow time of pure solvent, and *t* is the flow time of solution. Three measurements were performed on each sample, and the average value was calculated.

The number average molecular weight ($M\bar{n}$) of the samples was determined using the following Berkowitz Equation (2):

(2) $$M\bar{n}=3.29\times 10^{4}\;{\left[\eta \right]}^{1.54}$$

#### 2.4.4. Color Measurements

A Datacolor Spectraflash SF600 plus CT UV reflectance colorimeter (Datacolor, Marl, Germany) was used to measure color using the D65 illuminant and a 10° standard observer by excluding the UV component and including the specular component. Fivefold measurements were taken in each case using a unique holder (Datacolor), and the mean values were computed. The color of the PEF films was investigated according to the CIEL*a*b* color system. The L* axis measures luminosity or lightness ranging from 0 (black) to 100 (white), the a* coordinate measures redness when a is positive or greenness when a is negative, the b* coordinate measures yellowness when b is positive or blueness when b is negative, c* represents chroma, and H* represents hue angle. The K/S fraction was calculated to assess the concentration of the color on the PEF films [18].

#### 2.4.5. Differential Scanning Calorimetry

A PerkinElmer Pyris DSC-6 differential scanning calorimeter (PerkinElmer Inc., Massachusetts, USA) calibrated with pure indium and zinc standards was used. Samples of 6–8 mg sealed in aluminium pans were used, and all experiments were performed under an N_2_ atmosphere with a flow of 20 mL/min. The amorphous and annealed samples were first heated at 20 °C/min to 240 °C and held for 1 min. The samples were cooled with a cooling rate of 10 °C/min down to 30 °C and held for 1 min. The glass transition temperature (*T_g_*), melting temperature (*T_m_*), cold crystallization temperature (*T_cc_*), and enthalpies (*∆H_m_* and *∆H_cc_*) of the PEF samples were determined from these scans. Crystallinity degree (*X_c_*) was calculated with Equation (3):

(3) $$Xc_{\;}(\%)=\left(\frac{\Delta H_{m}-\Delta H_{cc}}{\Delta H_{m}^{0}}\right)\times 100$$

where $\Delta H_{m}^{0}$ *=* 137 J/g, as reported by our team in our previous work for the heat of melting of the 100% crystalline PEF.

#### 2.4.6. X-ray Diffraction

XRD was employed to study the semicrystalline structure of all samples at RT that had previously been melted and subsequently annealed (160 °C, 1 h). The XRD spectra were recorded using a MiniFlex II XRD system (Rigaku Co., Tokyo, Japan) with Cu Ka radiation (0.154 nm) over the 2θ range from 5° to 50° and with a scanning rate of 1°/min [19]. The % crystallinity was calculated from the XRD graphs using Equation (4):

(4) $$X_{c}={\left(1+\frac{A_{am}}{A_{c}}\right)}^{-1}$$

*A_am_* is the area of the amorphous halo, and *A_c_* is the area of the crystalline peaks.

#### 2.4.7. Broadband Dielectric Spectroscopy

BDS was employed for all samples to study molecular mobility via the recording of the complex dielectric permittivity, ε*(f, T) = ε′(f, T) − i·ε″(f, T), and evaluated the imaginary part (ε″) related to the dielectric losses. The measurements were conducted employing a Novocontrol BDS setup (Novocontrol GmbH, Montabaur, Germany) combined with a liquid nitrogen cooling system (Quatro, Novocontrol) on samples in the form of sandwich-like capacitors, with the polymer being melted at ~190 °C between finely polished cylindric brash electrodes. Silica spacers of ~100 μm were employed to prevent electrical contact between the electrodes, whereas, upon melting, the capacitors were cooled fast to keep the polymers in the amorphous state. The dielectric spectra were recorded in the fixed frequency range from 10^−1^ to 10^6^ Hz and within the temperature range from −150 to 180 °C, upon heating, at steps 5 and 10 K [20].

#### 2.4.8. Polarized Light Microscopy

PLM micrographs were recorded isothermally during melt crystallization. The micrographs were recorded using a Nikon Optiphot-1 polarizing microscope (Nikon, Tokyo, Japan) equipped with a Linkam THMS 600 heated stage, a Linkam TP91 control unit, and a Jenoptik Gryphax Arktur camera.

#### 2.4.9. Contact Angle

Contact angle measurements of all the PEF samples were measured using a goniometer from DataPhysics (Filderstadt, Germany). They were performed at room temperature with ultra-pure water (Millipore, Burlington, MA, USA) with a droplet volume of 3 μL, and an average value with standard deviation was calculated.

#### 2.4.10. Oxygen Permeability

The oxygen permeability was determined using Oxygen Transmission Rate System PERME^®^ OX2/230 (Labthink Instruments Co., Ltd., Jinan, China), by standard ASTM D3985-2005(2010) e1, ISO 15105-2:2003. Oxygen transmission rate (OTR) and coefficient values are average results obtained by two tests of five measurements. All specimens were conditioned at 23 °C and 50% relative humidity for 24 h before testing (flux = 10 mL/min). The thickness of PEF films was measured with a caliper at five different places. The films were tested using a specific mask to reduce the test area to 7 cm^2^.

#### 2.4.11. Nanoindentation Tests

Indentations were conducted using a dynamic ultra-micro-hardness tester (DUH-211; Shimadzu Co., Kyoto, Japan) fitted with a triangular pyramid indenter tip (Berkovich indenter, with a 100 nm tip radius). The indentations made on the surface of the investigated PEF samples appeared as an equilateral triangle. Ten measurements were conducted on each specimen, which was purposely scattered on the surface. After contact of the indenter with the surface, it was driven into the surface until a peak load of 20 mN was reached. The peak load was held for 3 s (to minimize the effect of viscoelastic deformation of the specimen, notably creep, on property measurements). Then, the indenter was unloaded to a load of zero [21].

## 3. Results and Discussion

### 3.1. Synthesis of PEF Starting from Different Monomers

The PEF polyesters were synthesized via melt polycondensation using two different furan-based monomers, FDCA (1:2.1 molar ratio) or its dimethyl ester DMFD (1:1.2, 1:1.5, 1:2.2 molar ratios). Generally, the synthesis of PEF is carried out in a high molar ratio due to the low solubility of FDCA in the diol. However, using large amounts of diols is not economically feasible from an industrial point of view as it requires high volumes of chemicals and the need for EG recycling. Additionally, using a higher number of diols results in increased diethylene glycol (DEG) content in the polymer chain, which affects the final properties of the sample [17]. Many published works have discussed this problem of ether-bridge formation during polymerization reactions [22,23]. For this reason, in all samples, molar ratios lower than 1/2.2 were used. The intrinsic viscosity, [*η*], ranges from PEF (FDCA) 0.43 to PEF (FDCA) 0.64 dL/g for PEF samples synthesized from FDCA and from PEF (DMFD) 0.28 to PEF (DMFD) 0.48 dL/g for PEF samples synthesized from DMFD. Furthermore, the average molecular weight ($M\bar{n}$), which was calculated using Equation (2), ranges from 4600 to 16,500, concerning both DMFD- and FDCA-derived polymers. The detailed sample list and synthetic conditions are displayed in Table 1. Sb_2_O_3_ was used as a catalyst for synthesizing PEF polyester using FDCA, while when PEF was synthesized starting from DMFD, zinc acetate, TBT, and zirconium(IV) isopropoxide were used as catalysts.

In a previous work by our group, Papadopoulos et al. investigated the catalytic activity of two industrial antimony catalysts, antimony trioxide (Sb_2_O_3_) and antimony acetate (Sb(CH_3_COO)_3_), in the two-step polymerization of FDCA with EG (1:3 molar ratio) to produce PEF polyesters [16]. It was found that Sb_2_O_3_ has higher activity than Sb(CH_3_COO)_3_. During the esterification step, the temperatures were 160, 170, and 190 °C for 4 h under nitrogen flow. Joshi et al. showed that the solubility of FDCA with diols is enhanced at temperatures ranging from 180–190 °C [24]. Additionally, FDCA was found to decompose at temperatures above 200 °C. Based on all these previous works, we decided to use esterification temperatures lower than 200 °C and, specifically, that ranged between 170 and 190 °C to increase the esterification rate and to avoid decomposition of FDCA, which would result in PEF samples with low molecular weight values. At these temperatures, the FDCA dispersion in EG became completely clear after 45 min, which is an indication that the esterification reaction was proceeding fast. Due to this observation, the esterification time was limited to 1.5 h, since due to a self-catalyzed reaction, the theoretically distilled H_2_O amount was already collected.

In the polycondensation step, as was found from a previous study [16], the reaction proceeds slowly at 220–230 °C; for this reason, in the present study, higher temperatures, such as 250–260 °C, have been used in order to accelerate the reaction and to prepare polyesters with larger molecular weights. Similarly, Sb_2_O_3_ (300 ppm) was used as an effective catalyst for polycondensation reactions. In the conditions used, it was found that by increasing the polycondensation time from 1.5 to 6 h, polyesters with higher intrinsic viscosity values that ranged from PEF (FDCA) 0.43 dL/g to PEF (FDCA) 0.64 dL/g were prepared. This is in good agreement with a previous work from Van Es et al., who reported that an increase in the molecular weight values of amorphous polymers under melt polymerization conditions is usually achieved by using high temperatures and extended reaction times [25].

In the case that DMFD was used as a monomer, a catalyst was needed from the beginning of the transesterification reaction, and the effect of different catalysts such as Zinc acetate (Zn(OAc)_2_), TBT, and zirconium(IV) isopropoxide on the IV increase was studied. It was found that the TBT catalyst (400 ppm) was more active, yielding PEF samples with intrinsic viscosity values from PEF (DMFD) 0.37 dL/g to PEF (DMFD) 0.48 dL/g, with a reducing DMFD/EG molar ratio from 1:2.2 to 1:1.2, respectively, as shown in Table 1. Additionally, an increase in the polycondensation time from 2.5 h to 6 h at 220–240 °C, increased the intrinsic viscosity value up to 0.48 dL/g. However, using FDCA as a starting monomer after 6 h of the polycondensation process led to an IV value of PEF (FDCA) equal to 0.64 dL/g versus 0.48 dL/g by using DMFD as a starting monomer, using the same polycondensation time. This indicates that FDCA could be the suitable monomer for the synthesis of high-molecular-weight PEF polyester.

Using Zn(OAc)_2_ as a catalyst to synthesize PEF polyesters from DMFD and EG at a 1:2.2 molar ratio, low MW (PEF (DMFD) 0.28 dL/g) has been also produced. The reaction time was increased in the first step until 5 h for the complete dissolution of DMFD into the diol. In the second polycondensation step, the temperature ranged between 230 and 250 °C, and the polycondensation time was 3 h, which was almost similar to that of the TBT catalyst (2.5 h). However, PEF with a lower IV value was produced, compared with TBT (PEF (DMFD) 0.37 dL/g). From these results, it seems that Zn(OAc)_2_ shows high activity for transesterification reactions but not for direct esterification reactions, which is in agreement with already published works [26,27,28]. Similar conditions have been used for zirconium(IV) isopropoxide catalysts. However, also in this case, PEF polyester with a low intrinsic viscosity PEF (DMFD) 0.32 dL/g was produced. Thus, it seems that TBT is a more effective catalyst compared with Zn(OAc)_2_ and zirconium(IV) isopropoxide since for the same DMFD/EG molar ratio 1:2.2 and similar used transesterification and polycondensation time (2.5 h) PEF (DMFD) 0.37 dL/g was produced (Table 1). Furthermore, in the case of the TBT catalyst, the used polycondensation time seems to also have an effect on the IV value of the produced PEF samples, while the DMFD/EG molar ratio does not. By increasing the polycondensation time from 2.5 to 4 h for the DMFD/EG molar ratio 1:1.5, PEF with IV values PEF (DMFD) 0.37 dL/g and PEF (DMFD) 0.45 dL/g were prepared, respectively. However, by increasing the polycondensation time to 6 h, only a small increase was achieved to PEF (DMFD) 0.48 dL/g.

The color of the synthesized PEF polyesters was also calculated using a color calorimeter and the colorimetric coordinates L*, a*, b*, C, h°, and K/S values are displayed in Table 2. It was found that the color ranges from light yellow to dark yellow, depending on different factors, such as altered polycondensation time (from 1.5 h to 6 h) and polycondensation temperature up to 260 °C in synthesizing PEF for both FDCA and DMFD monomers. This finding is in good agreement with the work from Qu et al., in which the author observed that the colorization of PEF samples became more severe with increasing polycondensation temperature and time, owing to the side reactions or polymer degradation [29].

Concerning the color of PEF samples produced using both FDCA and DMFD, it is clear that as the intrinsic viscosity increases, the L value decreases while a*, b*, and c* values increase, indicating a more saturated and yellowish color. The K/S value is an indication of the color concentration present in the sample: low K/S valued samples are bright yellow. The PEF samples with lower IV values appeared to have lower K/S values compared with the ones with higher IV, indicating that the increase in polycondensation time and temperature results in the yellowness of the sample.

An Sb_2_O_3_ catalyst used in the synthesis of FDCA samples results in higher L values and decreased a*, b*, c*, and K/S values for low IV of PEF (FDCA) 0.43 dL/g. Even at an increased IV to PEF (FDCA) 0.64 dL/g, it shows a strong increase in a*, b*, c*, and K/S values and decreased L values, which is due to the prolonged polycondensation time (6 h). This shows that Sb_2_O_3_ as a catalyst exhibited high activity and low coloration of the FDCA samples at short polycondensation time [16].

Among the metal catalysts used in the synthesis of DMFD samples, zinc acetate and zirconium(IV) isopropoxide exhibit higher L values and decreased a*, b*, c*, and K/S values compared with TBT catalysts. TBT-based DMFD samples (highest IV values) showed increased a*, b*, c*, and K/S values and decreased L values. It is rational that elevated temperatures and prolonged polycondensation time may result in the formation of colored furan-metal complexes. This finding is in good agreement with a previous work by Terzopoulou et al. The authors state that since it is known that furan dicarboxylic acid is a strong chelating agent, elevated polycondensation temperatures and prolonged polycondensation time can possibly explain the polymer discoloration and favor the formation of a metal–furan complex [26].

### 3.2. NMR and ATR Spectroscopy

**Figure 2:**
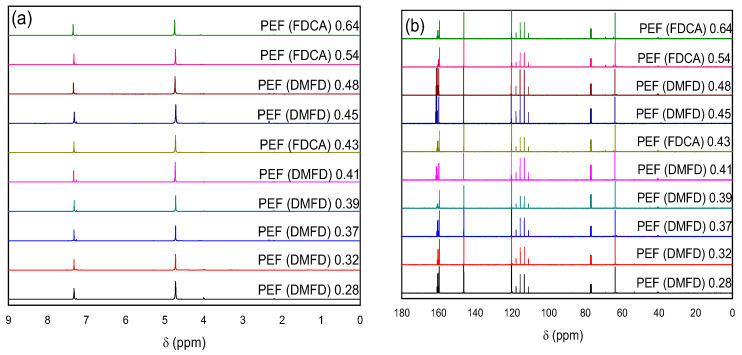
^1^H (**a**) and ^13^C (**b**) spectra of all synthesized PEF samples by increasing intrinsic viscosity. In the ^13^C spectra, the signals at approximately 40, 77, 111–117, and 160 ppm are due to the solvents used.

NMR and ATR spectroscopy were performed to identify the successful formation of PEF polyesters for both FDCA and DMFD samples. The NMR spectra of all PEF samples exhibit the expected resonance signals, confirming the successful polymerization for all samples, as shown in Figure 2.

Specifically, in the ^1^H-NMR spectra, two peaks are observed: at 4.72 ppm, corresponding to the –OCH_2_CH_2_O– protons, and at 7.32 ppm, corresponding to the CH aromatic protons of the furan ring. In the ^13^C-NMR spectra, the peak observed at 63.9 ppm is attributed to the –OCH_2_CH_2_O– carbon atoms, and the resonance signals at 120.3 and 146.3 ppm correspond to the carbon atoms of the furan ring. Finally, the ester C=O carbon atom gives a resonance at 159.6 ppm. It is noteworthy that, independently of the starting products or catalyst, polymerization was successful in all cases. PEF with IV values of PEF (FDCA) 0.43 dL/g, PEF (FDCA) 0.54 dL/g, and PEF (FDCA) 0.64 dL/g had a DEG content of 4%, 5%, and 6%, respectively, indicating that FDCA as a starting monomer or Sb_2_O_3_ as a catalyst could induce a higher DEG formation. For the other synthesized materials, the DEG content was lower but too low to be safely quantified.

**Figure 3:**
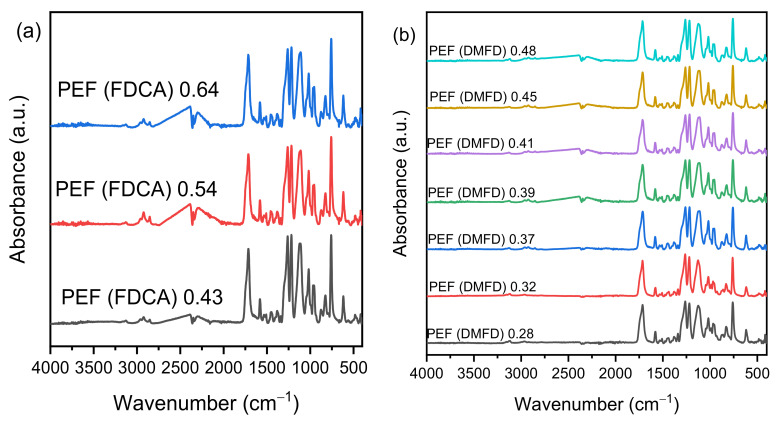
ATR spectra of the PEF samples (**a**) made from FDCA and (**b**) made from DMFD.

The ATR spectra of PEF samples made from FDCA are shown in Figure 3a: 3138–3159 cm^−1^ (C–H stretching vibrations of the furan ring); 2923–2931 cm^−1^, 2849–2873 cm^−1^ (asymmetric and symmetric C–H stretching vibrations); 1710–1717 cm^−1^ (C–O stretching vibrations); 1576–1584 cm^−1^ (aromatic C–C bending vibrations); 1506–1508 cm^−1^, 1451–1457 cm^−1^ (C–H deformation and wagging vibrations); 1374–1382 cm^−1^ (C–H rocking vibrations); 1261–1264 cm^−1^, 1221–1222 cm^−1^ (C–O stretching vibrations); 1106–1111 cm^−1^, 1012–1020 cm^−1^ ((C–O–C) ring vibrations, furan ring); 955–949 cm^−1^, 818–827 cm^−1^, 758–762 cm^−1^ (C–H out-of-plane deformation vibrations, furan ring); and 614–620 cm^−1^ (C–H bending vibrations) [30].

Figure 3b denotes the ATR spectra of PEF samples made from DMFD. All the DMFD samples have two weak bands near 3122–3160 cm^−1^ and 2938–2974 cm^−1^ that are related to out-of-plane and in-plane CH stretching of the furan ring, respectively, and a weak band around 2780–2844 cm^−1^ that is related to the out-of-plane stretching of alkyl methylene groups, a strong absorption peak related to the C=O stretching centered at 1719–1724 cm^−1^, and a band at 1554–1584 cm^−1^ linked to the C=C stretching vibration for all the samples. Similarly, an ester C–O stretching vibration was identified at 1256–1274 cm^−1^; a furan ring breathing band was identified at 1111–1134 cm^−1^, 1015–1024 cm^−1^; and ring bending was identified at 948–964 cm^−1^, 820–836 cm^−1^, and 758–763 cm^−1^. Both ATR spectra confirmed that polymerization was successful in all cases, regardless of the monomers, ratios, or catalyst.

### 3.3. Thermal Properties and Crystallinity

**Figure 4:**
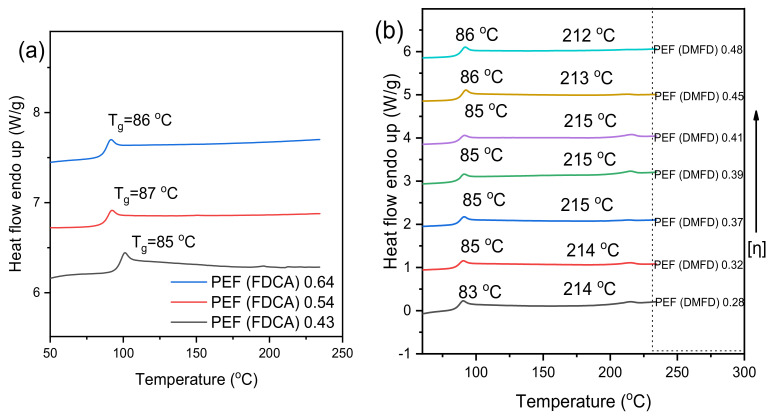
DSC scans (2nd heat, rate 20 °C/min) of (**a**) the PEF samples made from FDCA and (**b**) the PEF samples made from DMFD.

The thermal properties of the synthesized PEF samples were studied through DSC. Figure 4a,b present the second heating scans of amorphous FDCA and DMFD samples. The glass transition temperature (*T_g_*), melting temperature (*T_m_*), and cold crystallization temperature (*T_cc_*) values of PEF polyesters for both FDCA and DMFD samples are displayed in Table 3.

**Figure 5:**
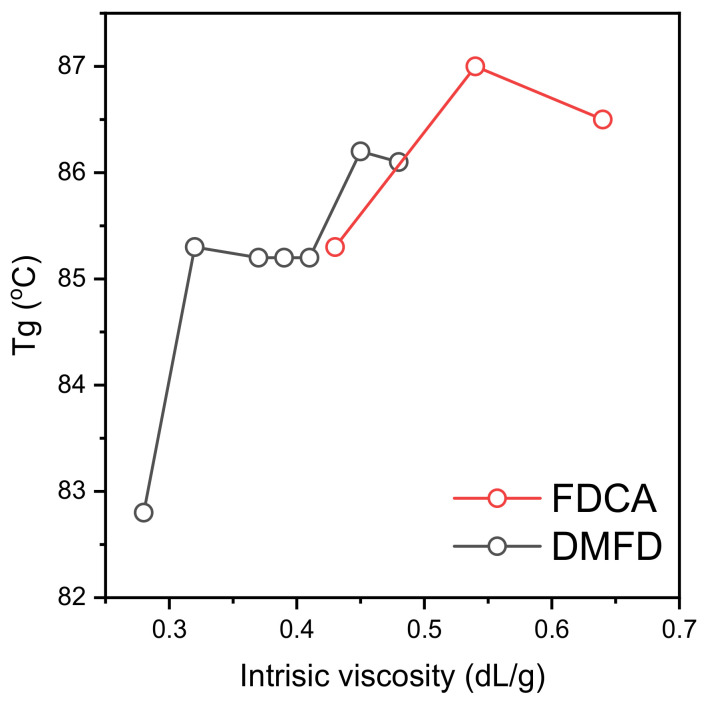
Effect of intrinsic viscosity on the glass transition temperature of PEF.

Terzopoulou et al. demonstrated that 2,5-FDCA is an excellent monomer for producing PEF polyesters with high *T_g_* [31]. The *T_g_* of FDCA samples increases from 85 to 87 °C with an increase in intrinsic viscosity from PEF (FDCA) 0.43 dL/g to PEF (FDCA) 0.54 dL/g. Similarly, the *T_g_* of DMFD samples increases from 83 °C to 86 °C for samples with increased IVs from PEF (DMFD) 0.28 dL/g to PEF (DMFD) 0.48 dL/g, respectively. The shifting of *T_g_* to higher temperatures is due to the chain entanglement caused by increased intrinsic viscosity [32]. Zubkiewicz et al. reported that furan-based polyesters have higher *T_g_* than PET due to decreased chain mobility and dipole–dipole interchain interactions of PEF samples [33]. Therefore, an increase in intrinsic viscosity leads to chain entanglement, and limiting the chain mobility increases the samples’ *T_g_* [34]. Further rises in polycondensation times and temperatures increase the intrinsic viscosity of the FDCA sample to PEF (FDCA) 0.64 dL/g. The decreased intermolecular interactions, as more FDCA were introduced into the polymer chain, make the polymer less cohesive and decrease the *T_g_* to 86 °C [35]. The effect of intrinsic viscosity on the glass transition temperature of FDCA samples is seen in Figure 5.

The absence of melting (*T_m_*) and cold crystallization (*T_cc_*) peaks in all FDCA samples indicates the long timescale of the crystallization process. The increased structural rigidity and nonlinear axis of rotation around the furan ring hinder the crystallization in all the FDCA samples [7]. However, in all DMFD samples, there is a melting peak recorded, which ranges between 212 and 215 °C. All the observed melting points T_m_ for the DMFD samples are well in agreement with those reported in the literature [23,36,37]. Cold crystallization temperature (*T_cc_*) is observed between 180 and 186 °C in the DMFD samples from PEF (DMFD) 0.28 dL/d to PEF (DMFD) 0.45 dL/g IV, due to high nucleus density [38]. Furthermore, an increase in the intrinsic viscosity of PEF (DMFD) 0.48 dL/g shows the absence of *T_cc_* due to increases in structural rigidity, which hinder the crystallization in the sample.

**Figure 6:**
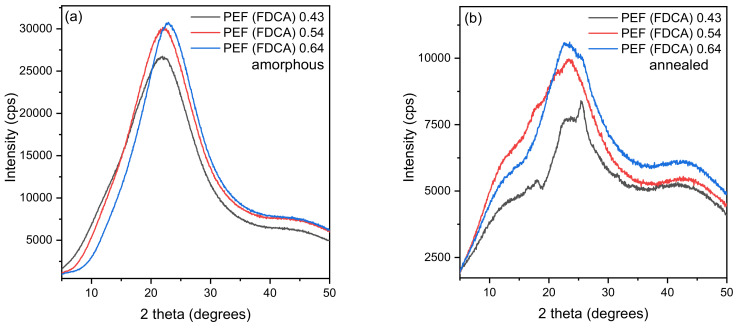

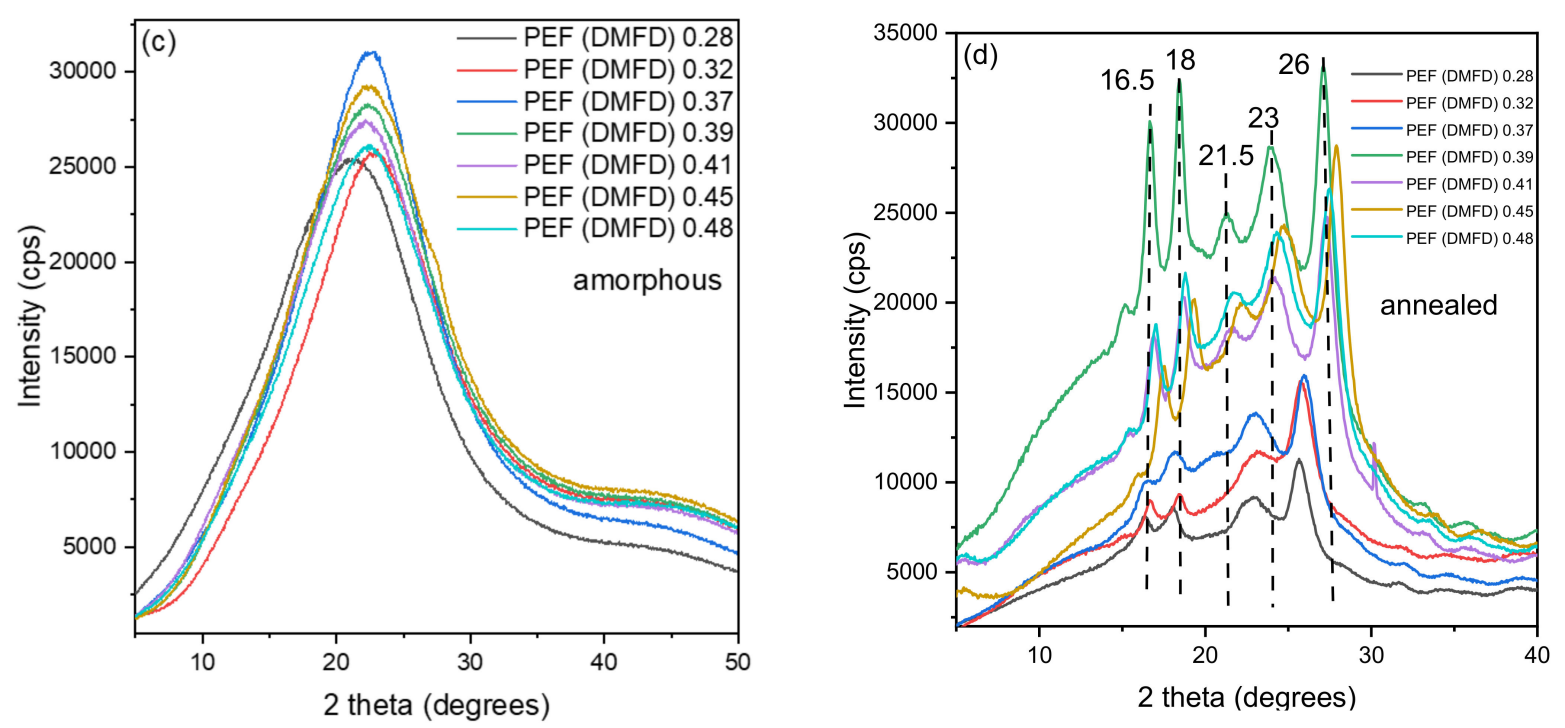
XRD patterns of amorphous and annealed PEF samples. Annealing at 160 °C for 1 h. **(a**) PEF-FDCA (amorphous), (**b**) PEF-FDCA (annealed), (**c**) PEF-DMFD (amorphous), and (**d**) PEF-DMFD (annealed).

XRD analysis was used to examine the crystalline structure of PEF samples produced from both monomers. Figure 6a,c display the XRD pattern of amorphous FDCA and DMFD samples, respectively. Figure 6a,c show an amorphous halo between 22.4° and 24.2° for all FDCA and DMFD samples, showing its amorphous nature. Based on the literature, PEF polyester has a lower degree of crystallinity due to the increased stiffness of the furan ring, and lower bond angles affect the hindering of furan ring motion [38]. Figure 6b,d displays the XRD pattern of annealed (160 °C for 1 h) FDCA and DMFD samples, respectively. After annealing, the FDCA sample with low intrinsic viscosity PEF (FDCA) 0.43 dL/g and PEF (FDCA) 0.54 dL/g showed less intense diffraction peaks at approximately 2θ ≈ 16.5°, 2θ ≈ 23°, and 2θ ≈ 26°, which indicates slight influence on crystallization. Tsanaktsis et al. studied the crystallization behavior of poly(ethylene furanoate) and observed that PEF (FDCA) 0.43 dL/g was recorded with three new peaks at 13.8°, 18.1°, and 26.7° after annealing at elevated temperatures [39]. The FDCA sample with a higher IV of PEF (FDCA) 0.64 dL/g only displays an amorphous halo centered around 2θ ≈ 22°. This is an indication that FDCA samples with higher intrinsic viscosities exhibit constrained chain mobility, which results in a slower crystallization rate. Additionally, the increased DEG content in FDCA samples damages the crystallinity of polyesters due to the mobility of the ether block [12]. From Figure 6d, all the annealed DMFD samples exhibit four main diffraction peaks around 2θ ≈ 16.5°, 2θ ≈ 18°, 2θ ≈ 23°, and 2θ ≈ 26°, which indicates the influence of crystallization in samples after annealing [38]. The samples synthesized using the TBT catalyst show high, intense peaks and crystallization with a new diffraction peak around 2θ ≈ 21.5°. From these results, it is observed that PEF samples synthesized using DMFD by the transesterification polycondensation method showed crystallization, which was attributed to low DEG content 1–2% [12]. The degree of crystallinity (*X_cb_*) of annealed samples calculated from XRD graphs were displayed in Table 4.

The degree of crystallinity (*X_c_*) for FDCA and DMFD annealed samples are analyzed from XRD graphs (Figure 6b,d) using Origin software. The *X_c_* was calculated from Aam and Ac using Equation (4) was displayed in Table 4. The FDCA sample with the lowest intrinsic viscosity of PEF (FDCA) 0.43 dL/g and PEF (FDCA) 0.54 dL/g had a very low degree of crystallinity (3%). The crystallization peaks were not observed for the FDCA sample with PEF (FDCA) 0.64 dL/g intrinsic viscosity. On the other hand, the DMFD sample with a low intrinsic viscosity of PEF (DMFD) 0.28 dL/g had the highest degree of crystallinity (35%), and the DMFD samples with the highest intrinsic viscosity of PEF (DMFD) 0.48 dL/g had the lowest degree of crystallinity (23%). According to these results, the degree of crystallinity (*X_c_*) tends to decrease with increasing intrinsic viscosity. This is due to an increase in the length of polymer chains; more tangled, constrained chain mobility; and significantly lower recrystallization rates [40].

**Figure 7:**
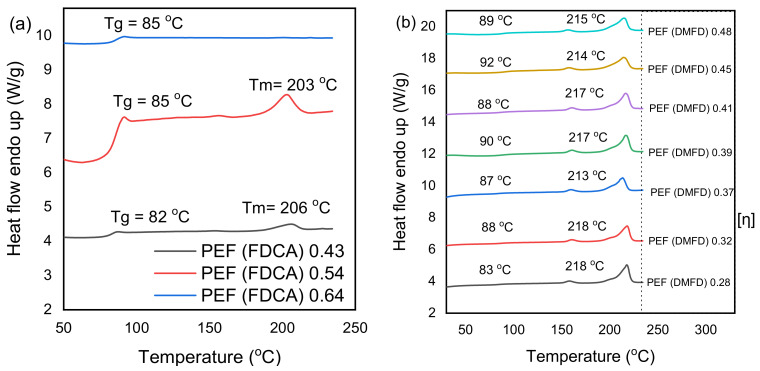
DSC scans after annealing (1st heat, rate 20 °C/min) of the (**a**) PEF samples made from FDCA and (**b**) PEF samples made from DMFD.

Figure 7a,b show the first heating DSC scans of annealed PEF samples (160 °C for 1 h) made from FDCA and DMFD with a heating rate of 20 °C/min. Thermal characteristics from the DSC scan after annealing are displayed in Table 4. It can be seen that the *T_g_* of PEF samples increases after annealing with increasing intrinsic viscosity. This is because the movement of the chains becomes more difficult as the molecular weight and % of crystallinity increases [39]. After annealing, two melting peaks, *T_m1_* and *T_m2_*, are observed in both FDCA and DMFD samples (Figure 7a,b) due to the melting of crystals of different morphologies with various thermal stabilities [33].

Stoclet et al. reported that the appearance of melting peak *T_m1_* at low temperatures, around 160 °C, is due to the secondary crystals melting because of the low-temperature crystallization. The observed melting peak *T_m2_* at higher temperatures is due to the melting–recrystallization–melting process [41]. Many works of the literature have reported the same melting behaviors of furan-based polyesters [42,43]. The lower recrystallization rate of annealed FDCA samples with increased intrinsic viscosity is due to the nonlinear nature of FDCA and the increased structural rigidity [7,40].

FDCA samples displayed a very low 2–3% crystallinity, indicating its amorphous nature, and DMFD samples have a % crystallinity ranging from 24 to 38%, as displayed in Table 4. The PEF (DMFD) 0.28 dL/g sample, which has the lowest intrinsic viscosity from all PEF samples prepared by DMFD, had the highest degree of crystallinity (38%). PEF sample 0.48, which has the highest intrinsic viscosity in the series of DMFD samples, had the lowest degree of crystallinity (24%). The degree of crystallinity (*X_c_*) tends to decrease with an increase in intrinsic viscosity due to the ensuing chain entanglement, resulting in constrained chain mobility and significantly reduced recrystallization rates, as observed in XRD thermograms [40]. Several works of the literature have discussed this phenomenon of recrystallization occurring before melting for thermoplastic polyesters [44,45].

### 3.4. Molecular Mobility and Ionic Conductivity (BDS)

The advanced technique of BDS was employed for the study of molecular mobility. We recorded the complex dielectric permittivity and evaluated its imaginary part of dielectric permittivity, ε″, which represents the dielectric loss [20].

**Figure 8:**
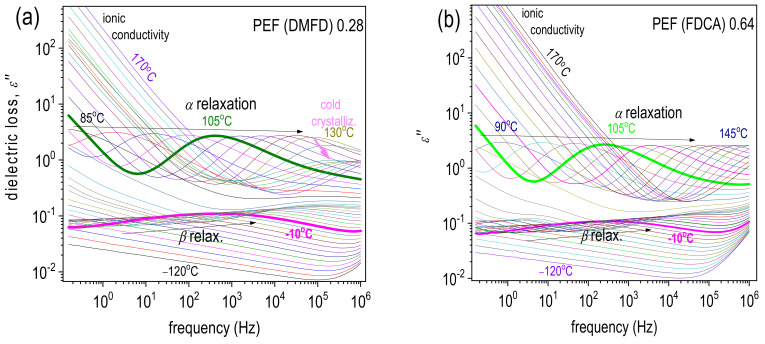
Isothermal curves of ε″ against frequency within the full temperature range of the BDS recordings for the two extreme cases of (**a**) PEF (DMFD) 0.28 dL/g and (**b**) PEF (FDCA) 0.64 dL/g. The resolved ε″ peaks, arising from local and segmental molecular motions, are marked for selected temperatures along with the corresponding temperature evolutions (added arrows).

Figure 8 shows representative results for the raw BDS measurements: the ε″(f) isothermal curves for the two extreme cases of PEF (DMFD) 0.28 dL/g and PEF (FDCA) 0.64 dL/g. Therein, the different groups of dipoles are responsible for the presence of individual ε″(f) ‘peaks’ upon the dipole’s relaxation. Such peaks are usually called ‘dielectric relaxations’, and their frequency position increases with the elevation in temperature. In contrast to calorimetry, the high resolving power of BDS enables the recording of local molecular motions (i.e., below the nanometric scale) along the nanometric scale, namely, the segmental mobility of the whole polymer chains.

**Figure 9:**
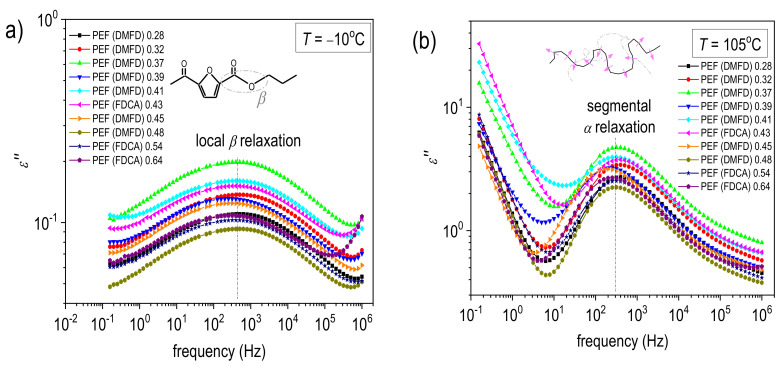
Comparative ε″(f) isothermal curves for all PEF samples at the selected temperatures of (**a**) −10 °C and (**b**) 105 °C to follow any effects on the local β and segmental α relaxation, respectively. The vertical dash-dotted lines in (**a**,**b**) were added to mark the relaxation peak positions. The inset scheme describes the molecular origins of the two processes.

In our case, two relaxations are recorded, the local β and the segmental α relaxation. Figure 9 presents comparative ε″(f) comparative for all samples at two selected temperatures, −10 °C and 105 °C, one focusing on β and the other on α relaxation.

**Figure 10:**
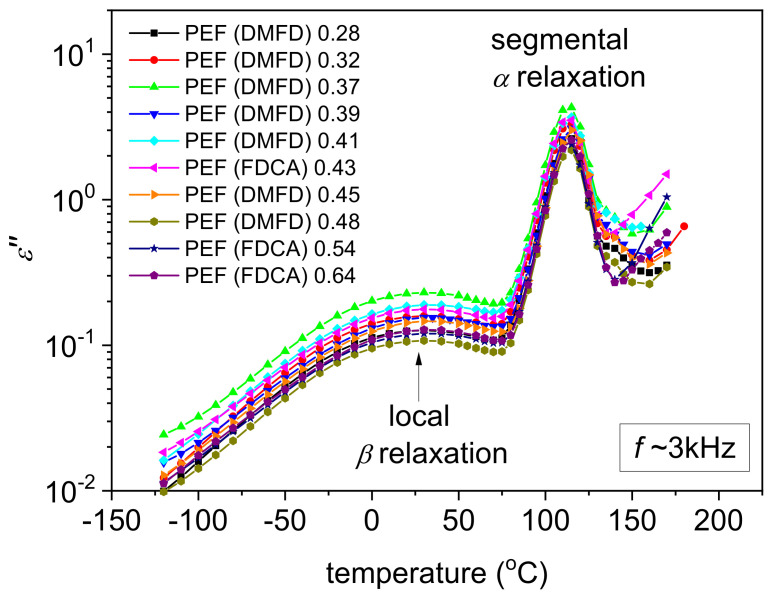
Comparative isochronal plots of ε″ against temperature, i.e., replottings of ε″(f), for all PEF samples at the frequency of 3.162 kHz. The main processes originating from molecular mobility are indicated in the plot.

In Figure 10, we show the same results in the form of isochronal ε″ curves, being drawn by the replotting of the isothermals at the selected frequency of ~3 kHz. Such representation enables an easier understanding of the temperature activation of the various molecular processes and a more direct comparison with the DSC heating traces. β relaxation has been proposed to arise from local crankshaft motions of the molecular group of PEF related to the chemical link between the ester carbon and the aromatic ring (inset scheme to Figure 9a). More details on and BDS results of PEF and various other poly(n-alkylene furanoate)s can be found in previous works [46,47,48,49,50]. Regarding the effects of the type of PEF studied here, no significant variations are recorded, neither in the frequency positions (time scale) nor in the strength of β.

At temperatures close to and above *T_g_*, the dielectric signal rises by more than one order of ε″ magnitude since the segmental relaxation gradually dominates. This relaxation screens the large-scale mobility of the polymer chains following the dipole moments, being perpendicular to the polymer chains backbone (inset scheme to Figure 9b). Thus, this is the so-called ‘main α relaxation’ and is the dielectric analog of the glass transition. As in the case of β, the effects on α by the PEF chain length or the different initial dicarboxylates (FDCA or DMFD) are quite weak. We recall that the BDS measurements were performed at initially amorphous samples. Thus, the effects of the polymer structure can be disentangled from those imposed by the presence of polymer crystals. So, we expect unaffected segmental mobility, considering the generally short PEF chains, which is confirmed by the recordings on α relaxation here.

In Figure 9b, we observe that for the very low molar mass PEF (DMFD) 0.28 dL/g, cold crystallization seems to take place at temperatures T ≥ 130 °C, as manifested by the sudden decrease in the magnitude of α relaxation [48]. The same also happens for the PEFs PEF (DMFD) 0.32 dL/g–PEF (FDCA) 0.43 dL/g, whereas not for the rest. This is in partial agreement with the results by DSC. However, cold crystallization is more intense in BDS, as it is facilitated due to the isothermal stay of the samples at each temperature for several minutes.

**Figure 11:**
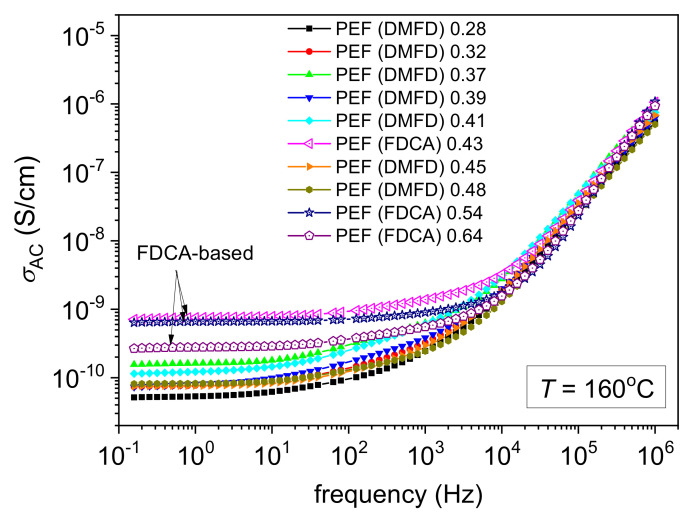
Comparative isothermal curves of the real part of conductivity (σAC or else σ′) for all PEFσ samples at a temperature selected significantly above *T_g_*, i.e., at 160 °C.

A final comment concerning BDS refers to ionic conductivity. This conductivity is recorded in the rubbery state of the polymer, i.e., above *T_g_*, and is due to motions of free charges (ions, impurities) throughout the whole sample’s volume. In ε″, the phenomenon is recorded as a quite sharp increase in the signal at T > *T_g_* and the lower frequency side of the measurement window (Figure 8). In terms of conductivity, σAC, a plateau is recorded (Figure 11), within which σ is independent of the frequency of the electric field. The absolute value of this plateau denotes the nature of conductivity, here being simply low and, thus, ionic. In Figure 11, three PEF samples exhibit significantly larger values for this plateau PEF (FDCA) 0.43 dL/g, PEF (FDCA) 0.54 dL/g, and PEF (FDCA) 0.64 dL/g. These are the PEFs synthesized using FDCA, whereas all those synthesized via DMFD exhibit lower σ plateau values. Although this is a secondary effect, it can be useful considering the small molecules permeation via PEF (oxygen, air, humidity) and can be exploited for certain applications, e.g., packaging.

### 3.5. Polarized Light Microscopy

**Figure 12:**
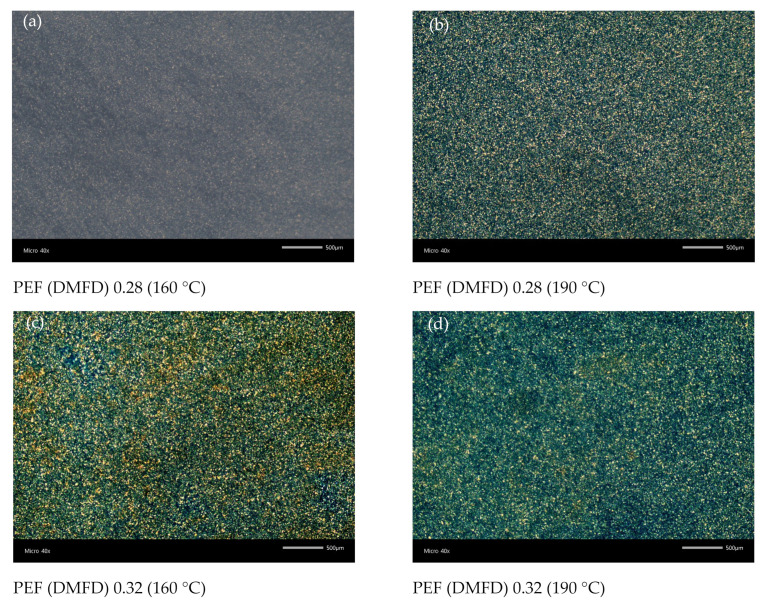

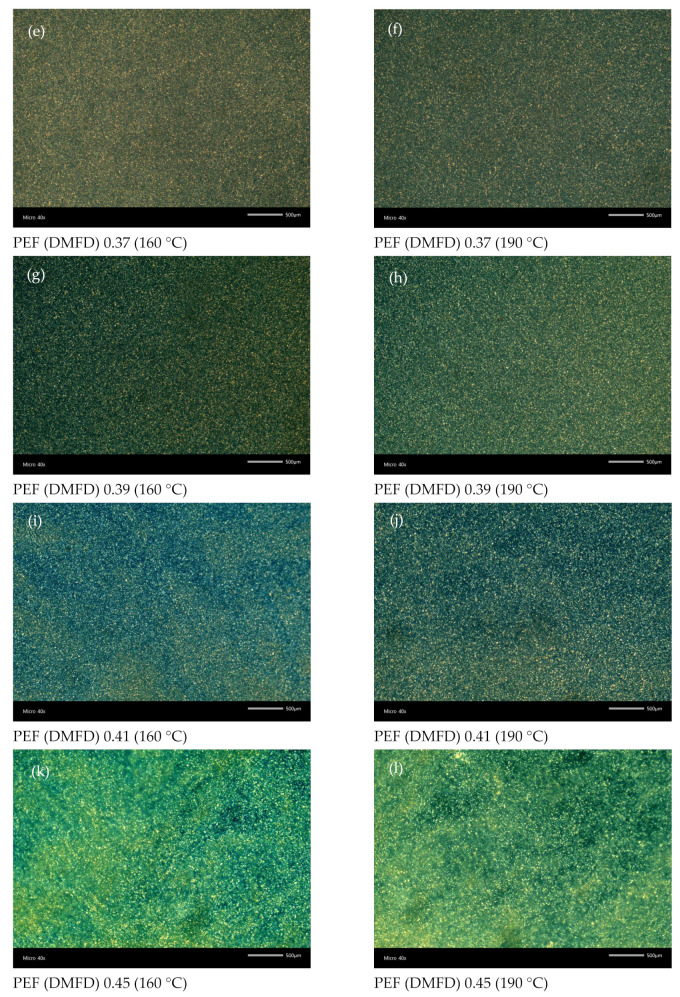

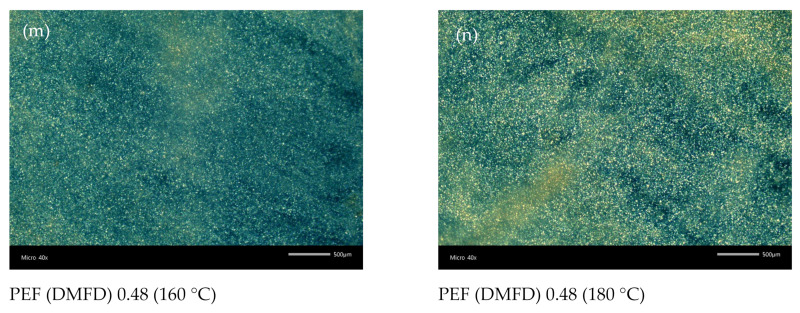
PLM after isothermal melt crystallization of DMFD samples with different intrinsic viscosities. (**a**) PEF (DMFD) 0.28 (160 °C), (**b**) PEF (DMFD) 0.28 (190 °C), (**c**) PEF (DMFD) 0.32 (160 °C), (**d**) PEF (DMFD) 0.32 (190 °C), (**e**) PEF (DMFD) 0.37 (160 °C), (**f**) PEF (DMFD) 0.37 (190 °C), (**g**) PEF (DMFD) 0.39 (160 °C), (**h**) PEF (DMFD) 0.39 (190 °C), (**i**) PEF (DMFD) 0.41 (160 °C), (**j**) PEF (DMFD) 0.41 (190 °C), (**k**) PEF (DMFD) 0.45 (160 °C), (**l**) PEF (DMFD) 0.45 (190 °C), (**m**) PEF (DMFD) 0.48 (160 °C), and (**n**) PEF (DMFD) 0.48 (180 °C).

The isothermal melt crystallization of PEF (DMFD) was followed with PLM at temperatures ranging from 160 °C to 190 °C. The magnification was ×40 for all the samples. In evidence from DSC and XRD, PEF samples obtained from FDCA required a long time for the crystallization process [7], making PLM images impossible to obtain. It is observed that the size of the spherulites starts increasing with increasing temperature from 160 °C to 190 °C. Similarly, Knoop et al. reported that at low temperatures, multiple nuclei exhibit small crystals, and at high temperatures, the nuclei density is limited, causing large crystals [36]. In comparison, the size of spherulites of PEF-DMFD samples was very small, which contrasts with thermoplastic polymers such as PET, poly(ethylene naphthalate) (PEN), poly (propylene terephthalate) (PPT), or poly (1,4-cyclohexanedimethylene 2,5-furandicarboxylate) (PCHDMF) [40]. The nucleation density of the PEF samples synthesized from DMFD increases with increasing intrinsic viscosity, as shown in Figure 12a–n. Unfortunately, PEF samples prepared by FDCA were not possible to be studied by PLM due to their slow crystallization rates, as was also observed by DSC.

### 3.6. Contact Angle and Oxygen Transmission Rate (OTR)

**Figure 13:**
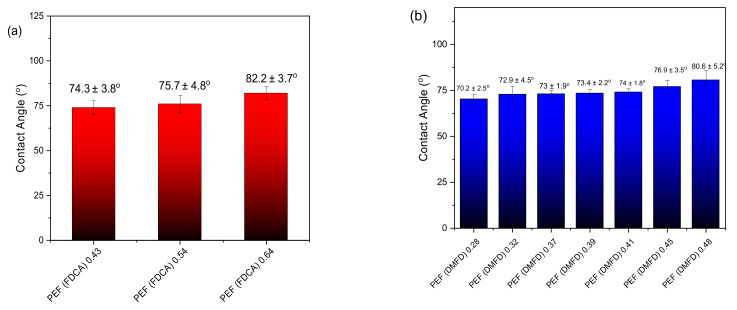
The average contact angle values of PEF samples with standard deviation and photography (**a**) from FDCA (**b**) from DMFD.

The wettability of the amorphous PEF films made from FDCA and DMFD samples measured by the surface contact angle technique at room temperature using melt-pressed films prepared at temperatures 20–30 °C above the melting point of each sample (Figure 13a,b). Quattrosoldi et al. and Ji et al. studied the structure of PEF and demonstrated that PEF is most likely to be hydrophilic due to the presence of oxygen atoms in the polymer backbone [51,52]. The contact angle values of FDCA samples increased from 74.3 ± 3.8° to 82.2 ± 3.7° with an increase in intrinsic viscosity from PEF (FDCA) 0.43 dL/g to PEF (FDCA) 0.64 dL/g. Similarly, the contact angle values of DMFD samples increased from 70.2 ± 2.5° to 80.6 ± 5.2° with an increase in intrinsic viscosity from PEF (DMFD) 0.28 dL/g to PEF (DMFD) 0.48 dL/g. This is because when increasing the intrinsic viscosity, the rigidity of the molecular chain increases too, and it decreases the surface hydrophilicity of PEF films [53]. Additionally, macromolecules with higher molecular weights have a lower number of –OH and –COOH end groups, which also leads to increased hydrophobicity. The differences between the PEF samples prepared using FDCA and DMFD are negligible.

Gas permeability is also very important for polyesters such as PEF since they are aimed to be used as food packaging materials. The average value of oxygen permeability with standard deviation is displayed in Table 5. Regarding increasing the intrinsic viscosities, the OTR values were reduced in the case of both FDCA and DMFD samples. The increase in polycondensation time and temperature evolves intrinsic viscosity and causes more rigidity of furan rings, hence lowering the oxygen permeability. Vannini et al. reported that the improved barrier performance of PEF compared with PET is due to the reduction in chain flexibility in the presence of furan rings [54]. The FDCA samples show a decrease in OTR values from 21.8 ± 4.9 to 1.6 ± 2.8 with an increase in viscosity from PEF (FDCA) 0.43 dL/g to PEF (FDCA) 0.64 dL/g. Similarly, DMFD samples show a decrease in OTR values from 25.4 ± 2.4 to 3.2 ± 0.9 with an increase in viscosity from PEF (DMFD) 0.28 dL/g to PEF (DMFD) 0.48 dL/g. So, it is clear that for the low oxygen permeability that is needed for food packaging applications, high-molecular-weight PEF samples should be synthesized.

### 3.7. Nanoindentation Test

**Figure 14:**
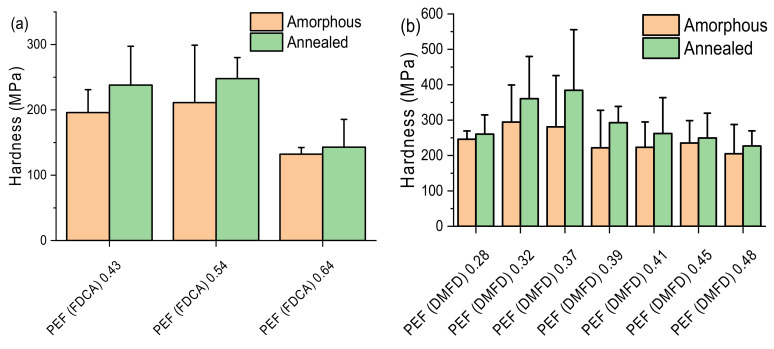
Comparative column diagrams for the hardness of amorphous and annealed samples PEF samples (**a**) made from FDCA and (**b**) made from DMFD.

The hardness indentations for both amorphous and annealed PEF samples from FDCA and DMFD are displayed as a comparative column diagram in Figure 14a and b, respectively. Balani et al. demonstrated that enhanced hardness, strength, and elastic modulus are observed due to more significant intermolecular bonding and the highly ordered lamellae of the polymers [55]. Similarly, the hardness of both FDCA and DMFD samples was higher in low to medium intrinsic viscosities due to shorter chains and lack of chain entanglement, making them more ordered lamella and increasing the hardness of the samples [34]. This is well in agreement with our findings since it is observed that increasing intrinsic viscosity increases the length of the polymer chain and decreases the intermolecular interactions due to the lower number of reactive groups. Hence, the hardness value is decreased, as seen in Figure 14a [33].

In the case of annealed FDCA samples and DMFD samples (160 °C for 1 h), the hardness of the annealed samples is higher compared with the amorphous samples due to the improved degree of crystallinity, which makes the samples more rigid. The hardness of the annealed samples tends to decrease with a decrease in the degree of crystallinity due to an increase in intrinsic viscosity. These results correlate with the degree of crystallinity (*X_c_*) values observed through XRD and DSC measurements.

**Figure 15:**
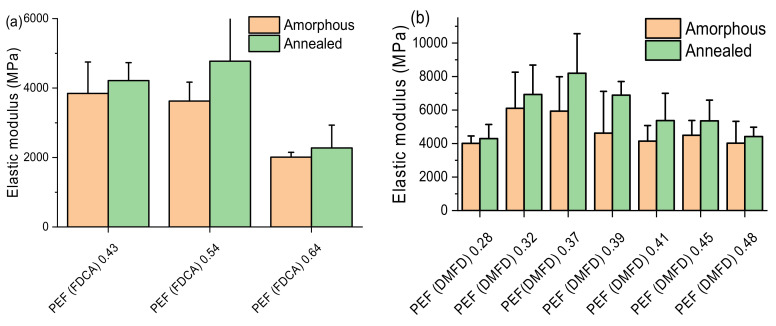
Comparative column diagrams for the elastic modulus of amorphous and annealed samples PEF samples (**a**) made from FDCA and (**b**) made from DMFD.

Gomes et al. analyzed the elastic modulus of PEF. They reported that, in general, PEF has a higher capacity to store energy, and hence, it is more rigid and probably more fragile [43]. The elastic modulus for both amorphous and annealed PEF samples from FDCA and DMFD are illustrated as a comparative column diagram in Figure 15a and b, respectively. As with hardness results, it is observed that amorphous FDCA and DMFD samples with low intrinsic viscosities tend to have high elastic modulus above 4000 MPa due to their shorter chains and high intermolecular interactions [34]. However, the further increase in the intrinsic viscosity value due to the formation of side chains results in decreased intermolecular interactions. This fact makes them less cohesive, causing a decrease in the samples’ elastic modulus of around 2000 MPa [33].

The elastic modulus of the annealed FDCA and DMFD samples is higher compared with the amorphous samples, which is due to the formation of crystals. [36]. However, further increasing the intrinsic viscosity values resulted in a lower degree of crystallinity. Hence, the elastic modulus decreased at a higher intrinsic viscosity in annealed FDCA and DMFD samples, as seen in Figure 15a,b.

From both hardness and elastic modulus results, it is observed that the FDCA samples had lower hardness and elastic modulus values compared with the DMFD samples. This may be due to the increased DEG content in the FDCA samples, as noticed in NMR spectroscopy. DEG units adversely affect the mechanical behavior, crystallinity, and barrier properties of the polyesters. During the synthesis of PET, DEG is used as a processing agent to soften its relatively rigid chain, lowering the crystallization of the polymer [12].

## 4. Conclusions

Bio-based poly(ethylene furanoate) was synthesized with different intrinsic viscosities using FDCA or DMFD as monomers with different molar ratios with EG, catalysts, reaction times, and temperatures. From intrinsic viscosities of PEF samples prepared with FDCA, it was found that they range from PEF (FDCA) 0.43 to PEF (FDCA) 0.64 dL/g, which is slightly higher when DMFD is used as monomer since their IV PEF samples range from PEF (DMFD) 0.28 dL/g to PEF (DMFD) 0.48 dL/g. This indicates that FDCA may be a better monomer to synthesize furanoate polyesters with high molecular weights. The color of the samples varied from light to dark yellowish brown, measured by colorimetry, dependent on the IV value and used catalyst type. The successful formation of PEF polyesters was confirmed using ATR-FTIR and NMR spectroscopies, and it was found that PEG is higher in FDCA samples. The thermal and crystallization properties of prepared samples were studied using DSC and XRD. It was found that the *T_g_* of amorphous PEF samples increased by increasing intrinsic viscosities due to chain entanglement and segmental mobility. Similarly, the annealed PEF samples showed a decrease in the degree of crystallinity (*X_c_*) with increasing intrinsic viscosity. PEF samples prepared by FDCA have much slower crystallization rates than those prepared by DMFD, which was also proved by PLM. The BDS results showed moderate effects on both the local and segmental dynamics, which was partly expected due to the generally low molar mass of all samples. However, the ionic conductivity varies at temperatures well above *T_g_*, which is enhanced for the PEF samples prepared using FDCA compared with those synthesized via DMFD. The wettability of PEF films recorded by contact angle measurements shows that hydrophilicity decreases with an increase in intrinsic viscosity due to an increase in the rigidity of the molecular chain and lower values of hydrophilic -OH and –COOH end groups. The OTR results show that the oxygen permeability of the samples decreases with an increase in the rigidity of the furan rings concerning intrinsic viscosities. The nanoindentation test showed that the hardness and elastic modulus of the amorphous PEF were higher at low to medium intrinsic viscosities due to shorter chains and high intermolecular interactions. The annealed PEF samples exhibit higher hardness and elastic modulus compared with amorphous samples due to an improved degree of crystallinity. In comparison with DMFD samples, FDCA samples have a lower hardness and elastic modulus due to the presence of increased DEG content.

## Figures and Tables

**Table 1:** Sample list and synthetic conditions.

Sample No	Monomers	Ratio	Time/Temperature (h/°C)		Catalyst (ppm)	[*ղ*] (dL/g)	*M_n_* (g/mol)
			1st Stage	2nd Stage			
1	FDCA:EG	1:2.1	1.5 h 170, 190–200	1.5 h 250–260	Sb_2_O_3_ 300	0.43	8900
2	FDCA:EG	1:2.1	1.5 h 170, 190–200	4 h 250–260	Sb_2_O_3_ 300	0.54	12,700
3	FDCA:EG	1:2.1	1.5 h 170,190–200	6 h 250–260	Sb_2_O_3_ 300	0.64	16,500
4	DMFD:EG	1:2.2	5 h 160–190	3 h 230–250	Zinc acetate 400	0.28	4600
5	DMFD:EG	1:2.2	4 h 160–190	2.5 h 220–240	TBT 400	0.37	7100
6	DMFD:EG	1:1.5	4 h 160–190	2.5 h 220–240	TBT 400	0.39	7700
7	DMFD:EG	1:1.5	4 h 160–190	4 h 220–240	TBT 400	0.45	9600
8	DMFD:EG	1:1.5	4 h 160–190	6 h 220–240	TBT 400	0.48	10,600
9	DMFD:EG	1:1.2	4 h 160–190	2.5 h 220–240	TBT 400	0.41	8300
10	DMFD:EG	1:1.2	3.5 h 160–190	3 h 240–250	Zirconium(IV) isopropoxide 400	0.32	5600

**Table 2:** Colorimetric data L*, a*, b*, c*, h°, and K/S values of the selected PEF samples.

Sample	Catalyst (ppm)	L*	a*	b*	c*	h°	R	K/S
PEF (FDCA) 0.43	Sb_2_O_3_ 300	90.07	−2.21	7.49	7.47	107.57	47.14 (400 nm)	0.30
PEF (FDCA) 0.64	Sb_2_O_3_ 300	80.27	−2.51	19.73	18.26	97.55	19.43 (400 nm)	1.67
PEF (DMFD) 0.28	Zinc acetate 400	88.48	−2.04	12.42	11.67	98.32	39.56 (400 nm)	0.46
PEF (DMFD) 0.32	Zirconium(IV) isopropoxide 400	85.30	−0.69	18.28	18.33	92.39	30.09 (400 nm)	0.81
PEF (DMFD) 0.37	TBT 400	83.46	1.2	22.56	24.45	90.24	26.29 (400 nm)	1.03
PEF (DMFD) 0.48	TBT 400	80.27	3.36	36.39	36.51	83.90	10.03 (400 nm)	4.03

**Table 3:** Thermal characteristics from the 2nd DSC scan.

Sample	*T_g_* (°C)	*T_cc_* (°C)	*T_m_* (°C)
PEF (FDCA) 0.43	85	-	-
PEF (FDCA) 0.54	87	-	-
PEF (FDCA) 0.64	86	-	-
PEF (DMFD) 0.28	83	182	214
PEF (DMFD) 0.32	85	183	214
PEF (DMFD) 0.37	85	183	215
PEF (DMFD) 0.39	85	186	215
PEF (DMFD) 0.41	85	185	215
PEF (DMFD) 0.45	86	180	213
PEF (DMFD) 0.48	86	-	212

**Table 4:** Thermal characteristics from the DSC scan after annealing (*Xc^a^* and *Xc^b^* are based on DSC and XRD measurements, respectively).

Sample	*T_g_* (°C)	*T_m_*_1_ (°C)	*T_m_*_2_ (°C)	*ΔH_m_ − ΔH_cc_* (J/g)	*Xc^a^* (%)	*Xc^b^* (%)
PEF (FDCA) 0.43	82	153	206	3	2	3.1
PEF (FDCA) 0.54	85	-	203	3	2	3.2
PEF (FDCA) 0.64	85	-	-	-	-	-
PEF (DMFD) 0.28	83	158	218	52	38	35
PEF (DMFD) 0.32	88	160	218	39	28	27
PEF (DMFD) 0.37	87	160	213	33	24	22
PEF (DMFD) 0.39	90	161	217	43	32	28
PEF (DMFD) 0.41	88	160	217	42	30	27
PEF (DMFD) 0.45	92	158	214	35	26	26
PEF (DMFD) 0.48	89	156	215	33	24	23

**Table 5:** The OTR values with a standard deviation of selected PEF samples.

Sample	Thickness of Films (µm)	STDV TR	OTR (cm^3^/m^2^d)
PEF (FDCA) 0.43	0.4	4.9	21.8
PEF (FDCA) 0.64	0.4	2.8	1.60
PEF (DMFD) 0.28	0.4	2.4	25.40
PEF (DMFD) 0.48	0.4	0.9	3.20

## Data Availability

No new data were created or analyzed in this study. Data sharing is not applicable to this article.
